# Counsellors contact dementia caregivers - predictors of utilisation in a longitudinal study

**DOI:** 10.1186/1471-2318-10-24

**Published:** 2010-05-14

**Authors:** Maria Grossfeld-Schmitz, Carolin Donath, Rolf Holle, Joerg Lauterberg, Stephan Ruckdaeschel, Hilmar Mehlig, Peter Marx, Sonja Wunder, Elmar Gräßel

**Affiliations:** 1 Psychiatric University Hospital Erlangen, Department of Medical Psychology and Medical Sociology, Schwabachanlage 6, 91054 Erlangen, Germany; 2 Helmholtz Zentrum München, Institute of Health Economics and Health Care Management, Ingolstädter Landstr. 1, 85764 Neuherberg, Germany; 3 Federal Association of the AOK, Rosenthalerstr. 31, 10178 Berlin, Germany; 4 HealthEcon AG, Basel, Switzerland; 5 Eisai GmbH, Lyoner Str. 36, 60528 Frankfurt, Germany; 6 Pfizer Deutschland GmbH, Linkstrasse 10, 10785 Berlin, Germany; 7 AOK Bavaria - Health insurer, Stromerstraße 5, 90443 Nürnberg, Germany

## Abstract

**Background:**

Counselling of family members is an established procedure in the support of dementia patients' relatives. In absence of widespread specialised dementia care services in most countries, however, counselling services are often not taken up or only very late in the course of the disease.

**Object:**

In order to promote acceptance of this service, a new counselling concept was implemented where general practitioners recommended family counsellors, who then actively contacted the family caregivers to offer counselling ("Counsellors Contact Caregivers", CCC). The research questions were: To what extent can the rate of family counselling be increased by CCC? What are the predictors for usage of this form of family counselling?

**Methods:**

The study started in June 2006 in Middle Franconia for patients with mild to moderate dementia. At baseline, 110 family caregivers were offered counselling based on the CCC guideline. Data was analysed from 97 patient-caregiver dyads who received counselling for one year. The mean age of the patients with dementia (67 women and 30 men) was 80.7 years (SD = 6.2). The mean age of their primary family caregivers (68 women, 23 men) was 60.8 years (SD = 13.8).

**Results:**

35 family members (36%) made use of more extensive counselling (more than one personal contact). By contrast, 29 family members (30%) had no personal contact or only one personal contact (33 cases, 34%). The factors "spouse" (p = .001) and "degree of care" (p = .005) were identified as significant predictors for acceptance of extensive counselling.

**Conclusions:**

Actively contacting patients and their caregivers is a successful means of establishing early and frequent contact with family members of patients with mild to moderate dementia. Use of extensive counselling is made especially by spouses of patients requiring intensified care.

**Trial Registration:**

ISRCTN68329593

## Background

Caring for a patient with dementia at home demands considerable adaptive behaviour from the family caregiver [1,2]. The family caregiver must learn to deal with cognitive deficits, disorientation and changes in the dementia patient's personality and behaviour. For this reason, more than one-third of the primary family caregivers feel heavily to extremely burdened [3]. This results in a clear deterioration in the emotional and physical health of the family caregiver [1,4]. Systematic counselling of family caregivers offers direct relief through conversations. Moreover, the family members are informed of additional relief offers. However, the degree of usage, according to international studies, is only between 4% and 31% [5-9]. In a cross-sectional study in Germany, Schneekloth found that 16% of family caregivers of patients with dementia use telephone counselling and less than 10% make use of personal contacts between family members and family counsellors [10].

The reason for the rather low use made of family counselling is primarily that early in the disease, the estimate of how the disease will develop is unrealistic and there is incomplete information about the offers available. Thus, contact with a counselling service often is only made when the disease is in an advanced stage [11,12]. Prevention of deterioration of the family caregiver's health due to stress can hardly be effective any longer. The utilization situation is decisively influenced by the fact that the family caregiver usually must take the initiative in establishing contact with a family counselling service [13]. Although there are many different concepts of counselling for caregivers and case management projects, the counsellors rarely initiate either a preventive approach or active contact with the caregivers [9,14]. Therefore the concept of Counsellors Contact Caregivers (CCC) was developed, in order to reach more family caregivers, on the one hand, and to provide earlier counselling on the other. This is important since information about support services is often unavailable or obtained too late [11,15]. Now that CCC has been tested in a pilot study for its manageability [13], the results reported here are part of the first randomised controlled intervention study in Germany incorporating the concept of family counsellors who contact caregivers of patients with dementia as a core element.

The present article first presents the guideline-based concept of CCC, and then addresses the following empirical research questions: To what extent can the rate of family counselling be increased by CCC? What are the predictors for usage of this form of family counselling?

## Methods

### Design

The concept of CCC is part of the intervention in the cluster-randomised controlled study "IDA". IDA is the German abbreviation of "Dementia Care Initiative in Primary Practice". The study protocol was published [16]. The study region was Middle Franconia, which is a mixed urban-rural area around Nuremberg in South-East Germany [16]. The IDA-study started in June 2006 and ended in December 2008. General practitioners were randomized in three intervention groups and all patients and family caregivers of these general practitioners were therefore gathered in one of these three groups in cluster-randomization. In two intervention groups, two non-medical interventions were compared to a control group with respect to cost and outcome. Control group A (171 patients) got usual care whereas in the two intervention groups B (109 patients) and C (110 patients), GPs recommended support groups (B and C) and offered family counselling beginning at baseline (only in C). The primary endpoint in the IDA-study was the patient's death or institutionalization. The special feature of IDA is that the general practitioner (GP) arranged additional support initiatives for the family caregivers. Usage of the additional offers was, however, voluntary and not requisite for study participation. Four counsellors offered family counselling following a newly-developed guideline ("Counsellor Contact Caregivers" - see Additional file 1, Table S1). The results presented here are taken from intervention group C, since CCC was applied beginning at baseline in this group.

The inclusion criteria for patients were: MMSE [17] score between 10 and 24 points, age ≥ 65 years, support by a family caregiver, living at home and member of a specific health insurance company (AOK). Patients with nursing home placement planned in the short-term or a shortened life expectancy of less than six months and those unable to give written informed consent were excluded. The data used here are medical examination data and data from telephone interviews with family members at baseline. The counsellors provided anonymous information about the general counselling contents. In order to protect the personal confidential relationship between the counsellor and caregiver, the following case-related data were not available for analysis: Resources and deficiencies within the families as well as detailed content and aims of counselling; individual risk factors for terminating care at home and the measures individually recommended by the counsellor (termed "protected data" in the informed consent).

The study was reviewed by the Ethics Committee of the Bavarian Chamber of Physicians (Date of approval: 30/05/2005, Reference number 05029) and is in compliance with the Helsinki Declaration. Signed informed consent was obtained from all patients and caregivers.

### Intervention - Concept of Counsellors Contact Caregivers (CCC)

In order to meet the goals of patients and family members, who usually wish the patient to live at home for as long as possible; the family counsellors were trained with respect to the risk factors for terminating home care.

The major risk factors for institutionalisation were identified based on scientific literature. Afterwards, guidelines were defined in an expert group (three researchers in cooperation with the counsellors) on how to react if certain risk factors became evident (see Additional file 1, Table S1).

In absence of widespread specialised dementia care services in Germany, the GP is the primary contact person and source of information about further support services for patients with dementia and their families. Therefore it is important that he/she recommends counselling. The main intention of the counselling concept is to give the family caregivers information about the patient's situation, availability of help as well as psycho-educational support.

The four counsellors in the IDA project, who actively approached the family every 6 to 8 weeks by telephone, used elements of case management, when personal contact was established. The counselling process consisted of assessment, target agreement, intervention, monitoring and evaluation. The counsellors were available during normal working hours and offered calls to their clients' homes. They were state-registered nurses or nurses trained in the care of the elderly and all have several years experience in psychogeriatric care. At the beginning of the project, the counsellors participated in training sessions on communication and social insurance code. The counsellors were to attempt to make at least one house call or one personal contact in order to assess the care situation. As customary in other counselling processes, the first personal contact served primarily to get to know one another and to record health status and care needs [14,18,19]. Further personal contacts followed when, during the first contact, a sort of "counselling order" was established so that more extensive counselling contacts could be made. With regard to the contents of the counselling sessions, special attention was to be paid to possible risk factors, as described in the CCC guideline, (see Additional file 1, Table S1).The contents were documented in very generalized form for the scientists. The topic areas included: "Patient's physical situation" (General physical status, development of the disease, comorbidities of the patient), "Caregiver's physical situation" (General physical status of the caregiver), "Emotional situation of the patient" (Emotional issues concerning the patient), "Emotional situation of the caregiver" (Emotional issues concerning the caregiver), "General framework" (i.e. provision of medical and other supportive aids, suitable adaptation of housing, financial situation), "Caregiving activities" (i.e. information on ADL, IADL, supervision), "Social support" (i.e. Contact with friends and family or other supportive measures) and "Additional topics of the caregiver" (i.e. Further commitments of the caregiver, for example caregiving for further persons).

The unique characteristic of CCC is that approaching family members should enable earlier planning of assistance than is the case in the "usual" family counselling programs, which can only become active once the family members themselves seek to establish contact.

### Measurements

The patient's medical file contains the following data: Sociodemographic data, severity of dementia, measured with the Mini-Mental Status Examination (MMSE) [17], secondary symptoms of dementia, and comorbidities.

Statements were obtained from family members using a computer-assisted telephone interview. These included: Sociodemographic data, Barthel Index [20], to measure patient's functional independence, two subscales of the Nurses' Observation Scale for Geriatric Patients NOSGER (subscale IADL, instrumental activities of daily living, and subscale disturbing behaviour) [21], the Burden Scale for Family Caregivers (BSFC) [3] to assess caregiver's subjective burden and an extended version of the questions on informal care time of the Resource Utilization in Dementia instrument (RUD) [22,23].

The following data were provided by the counsellors: date, duration and method of contact (telephone call, personal visit, home visit, written contact, contact with institutions, contact with GP) and up to three main conversation partners (i.e. caregiver, patient). In addition, up to eight much generalized topic areas were documented which were addressed in the contacts between counsellors and family caregivers (see section Intervention). These topic areas were divided into: "Patient's physical situation", "Caregiver's physical situation", "Emotional situation of the patient", "Emotional situation of the caregiver", "General framework, "Caregiving activities", "Social support" and "Additional topics of the caregiver".

### Statistical Analysis

For the statistical analysis, Statistical Package for Social Sciences (SPSS^® ^16.0) was used. T-tests were used to analyse group differences in the case of continuous variables; Chi-square tests in the case of categorical variables. In order to reveal the relational structure of the 17 potential predictor variables and thus reduce collinearity-related problems during data analysis in a bivariate logistic multiple regression analysis, a factor analysis was first performed using the main component method with subsequent orthogonal rotation.

Five factors were extracted, which were then entered in a binary-logistic regression analysis as independent variables to determine the predictors for usage of extensive counselling. The dichotomous dependent variable was taking advantage of more than one personal contact ("extensive counselling", code 1), or no or only one personal contact (code 0). A p-value < 0.05 was defined as level of significance.

A sensitivity analysis including several interaction terms in the original model was performed. The interaction terms turned out to be statistically insignificant: Factor-1 × Factor-2 (p = .909); Factor 2 × Factor 5 (p = .500), Factor 3 × Factor 4 (p = .161). Therefore the main-effect model seems to be adequate.

## Results

In intervention group C, 110 family caregivers were offered CCC at baseline. Data was analysed from 97 patient-caregiver dyads (total sample), because two patients or their caregivers refused to participate in the study any further and one caregiver looks after two patients. We excluded those patient-caregiver dyads where patients died or entered nursing home within the first six month of the intervention (n = 10), as these patient-caregiver dyads could not participate in further counselling contacts and would therefore be a bias to the description of the counselling process. In the case of one caregiver who looks after two patients, only the patient with the lower MMSE score was taken into account.

The mean age of the patients with dementia (67 women and 30 men) was 80.7 years (SD = 6.2). The mean age of their primary family caregivers (68 women, 23 men) was 60.8 years (SD = 13.8). Further characteristics of the sample are given in Table 1.

**Table 1:** Characteristics of patients and caregivers

Variables	Total sample (n = 97)	Subgroup I: No or only one personal contact (n = 62) Mean (SD)/n (%)	Subgroup II: More than one personal contact (n = 35) Mean (SD)/n (%)	p
Patient's age	80.7 (6.2)	80.8 (5.8)	80.5 (6.9)	.861
Caregiver's age*	60.8 (13.8)	56.9 (13.1)	67.6 (12.6)	**< .001**
Patient's sex (female)	67 (69%)	46 (74%)	21 (60%)	.146
Caregiver's sex (female)*	68 (70%)	47 (81%)	21 (64%)	.066
Employed caregiver*	31 (32%)	27 (47%)	5 (15%)	.**001**
Relationship (caregiver to patient)***: Spouse Son/Daughter (-in-law) Others	33 (34%) 47 (49%) 12 (12%)	15 (25%) 36 (61%) 8 (14%)	18 (55%) 11 (33%) 4 (12%)	**.016**
Hours spent on ADLs (RUD)**	1.8 (2.1)	1.25 (1.7)	2.7 (2.4)	.**004**
Hours spent on IADLs (RUD)***	2.0 (2.1)	1.5 (1.6)	2.7 (2.5)	.**017**
Hours spent supervising patient activities (RUD)*	2.1 (3.7)	1.4 (3.5)	3.3 (3.9)	**.022**
Subjective Burden (Burden Scale for Family Carers)**	24.4 (16.6)	22.2 (17.5)	38.4 (14.4)	.088
Functional independence (Barthel Index)**	70.1 (28.6)	74.2 (27.4)	62.5 (29.6)	.062
Cognitive decline (MMST)	18.5 (3.9)	19.3 (3.8)	16.9 (3.7)	.**003**
Instrumental activities of daily living (NOSGER subscale IADL)***	15.8 (5.8)	15.1 (5.6)	17.0 (6.1)	.135
Disturbing behaviour (NOSGER: subscale disturbing behaviour)*	9.6 (3.7)	9.1 (3.7)	10.4 (3.7)	.095
Number of comorbidities	3.2 (1.5)	3.5 (1.5)	3.2 (1.5)	.566
Presence of psychiatric symptoms+ (depression, anxiety, delusion)	38 (39%)	27 (44%)	11 (31%)	.240
Presence of behavioural symptoms+ (aggression, roaming, insomnia, agitation)	58 (60%)	38 (61%)	20 (57%)	.689

*Valid cases: Total number n = 91/Subgroup I n = 58/Subgroup II n = 33

**Valid cases: 91/59/32

***Valid cases: 92/59/33

+ One or more symptoms present

Families were contacted by the counsellors primarily by telephone and within one year after the GP's recommendation. "Personal contact" is defined as face-to-face contact (at client's home or elsewhere). In the total sample 29 family members (30%) had no personal contact and 33 family members (34%) had only one personal contact (subgroup I), while 35 family members (36%) made use of more extensive counselling (more than one personal contact; subgroup II). Overall, the first personal contact was made within the first 3 months after arrangement by the GP. 82% of the cases were house calls. In 18% of the cases, the caregiver and counsellor met elsewhere.

**Figure 1:**
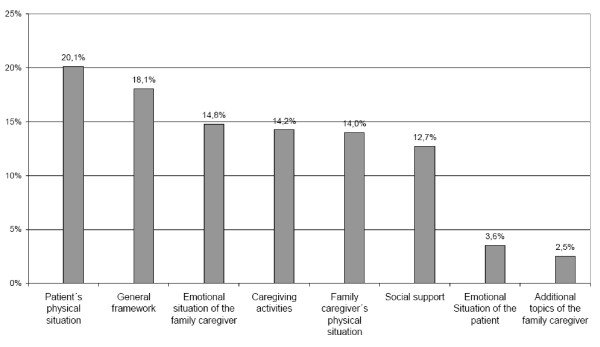
**Main topics in more extensive counselling contacts**. Patient's physical situation: General physical status, development of the disease, comorbidities of the patient/General framework: Provision of medical and other supportive aids, suitable adaptation of housing, financial situation of patient and caregiver/Emotional situation of the family caregiver: Emotional issues concerning the caregiver/Caregiving activities: information on ADL, IADL, supervision/Family caregiver's physical situation: General physical status of caregiver/Social support: Contact with friends and family or other supportive measures/Emotional situation of the patient: Emotional issues concerning the patient/Additional topics of the family caregiver: Further commitments, caregiving for further persons

The 35 family members (36%) of subgroup II met with the counsellor on average (mean) 3.9 times within a period of 12 months (range 2-10 contacts). The most frequent topics were the patient's physical situation (20.1%), followed by general framework (18.1%) (i.e. provision of medical and other supportive aids, suitable adaptation of housing, financial situation) (see Figure 1).

The two subgroups differ significantly in the way that in subgroup II (extensive counselling) the family members are older, less often employed, more often the spouse of the dementia patient, spend more time for all aspects of care at home, and that the dementia patient is cognitively more impaired (Table 1). We used a two step procedure to identify predictors for usage of more extensive counselling: First a factor analysis was performed since several of the 17 recorded variables showed strong correlation. This resulted in five factors with an inherent value greater than 1.0 (extraction criterion): Factor 1 (inherent value 4.4/26% explained variance), Factor 2 (2.8/17%), Factor 3 (1.6/9%), Factor 4 (1.6/9%), and Factor 5 (1.5/9%). Together the five extracted factors explain 70% of the total variance.

**Factor 1 (see Table **2**): **Main variable: Subjective burden of the caregiver. It shows a positive correlation with hours spend on activities of daily living (ADL), IADL, supervision, NOSGER subscale IADL and subscale disturbing behaviour and a negative correlation with the Barthel Index and MMSE. This means that subjective burden increases when the patient needs more extensive care due to his decreasing physical status. This factor thus represents the area "**Objective and subjective burden of care"**.

**Table 2:** Loading of the variables in the factor analysis

Variables	Factors				
	1	2	3	4	5
Patient's age	.200	.058	**.712**	-.281	-.142
Caregiver's age	.022	**.915**	-.005	-.120	-.090
Patient's sex*	.028	.429	-.179	-.044	**.765**
Caregiver's sex*	-.107	.276	-.077	.149	**-.810**
Employed caregiver+	-.152	**-.789**	-.044	.213	.081
Relationship (caregiver to patient)#: Spouse Son/Daughter (-in-law) or Others	.012	**.879**	-.238	.157	.213
Hours spent on ADLs (RUD)	**.767**	.287	.129	.106	.215
Hours spent on IADLs (RUD)	**.560**	.379	.215	.239	.202
Hours spent supervising patient activities (RUD)	**.700**	.078	-.144	-.237	.038
Subjective burden (Burden Scale for Family Carers)	**.838**	-.035	-.028	-.003	-.039
Functional independence (Barthel Index)	**-.731**	-.059	-.337	-.292	-.168
Cognitive decline (MMST)	**-.593**	-.092	-.043	-.410	.066
Instrumental activities of daily living (NOSGER subscale IADL)	**.770**	-.023	.196	.196	.159
Disturbing behaviour (NOSGER subscale disturbing behaviour)	**.774**	-.074	-.189	.056	-.213
Number of Comorbidities	-.087	-.174	**.818**	.052	.057
Presence of psychiatric symptoms (depression, anxiety, delusion)+	-.004	-.056	-.210	**.748**	-.143
Presence of behavioural symptoms (aggression, roaming, insomnia, agitation)+	.315	-.128	.048	**.637**	-.013

|Loading| ≥ .05 in bold type

* 1 = male, 0 = female

+ 1 = yes, 0 = no

# 1= spouse, 0 = Son/Daughter (-in-law)/Others

**Factor 2: **Main variable: Caregiver's age. Age correlates positively with spouse-relationship and negatively with employment. This factor represents the **"Spouses" **who are older and therefore more often unemployed than the children/childrens' spouse caregivers.

**Factor 3: **Main variable: Number of comorbidities. It shows a positive correlation with the age of the patient. This factor represents the area **"Comorbidities"**.

**Factor 4: **Main variable: Presence of psychiatric and behavioural attendant symptoms. This factor represents the **"Attendant symptoms"**.

**Factor 5: **Main variable: Caregiver's sex. The negative correlation with patient's sex means that male patients with dementia are more often cared for by women. This factor represents the "**Gender ratio"**.

Second a binary-logistic regression analysis was performed with the five factors as independent variables and usage or non-usage of more extensive counselling as dependent variable (Table 3). It resulted in a significant model (χ^2 ^(5) = 22.970; p < 0.001) results for the. The model has an explained variance of 31.5% (R²) and identifies two significant predictor variables. With this, 72.7% of the cases could be correctly ascribed to the categories "Usage of extensive counselling" or "Non-usage of extensive counselling". The probability of usage of more extensive counselling is significantly greater when the patient is the spouse (Factor 2) or when the subjective and objective burden of care is elevated (Factor 1).

**Table 3:** Results of the binary-logistic regression analysis

	Regression coefficient β	Standard error	Wald coefficient	df	Sig.
Factor 1: **Objective and subjective burden of care**	.731	.258	8.004	1	**.005**
Factor 2: **Spouses**	1.004	.293	11.765	1	**.001**
Factor 3: **Comorbidities**	.037	.246	.023	1	.879
Factor 4: **Attendant symptoms**	-.236	.257	.846	1	.358
Factor 5: **Gender ratio**	-.244	.251	.948	1	.330

How to predict utilization of counselling utilization for a new case:

The probability for using counselling can be computed by

e = Euler's constant

while z = Factor1 × β_Factor1 _+ Factor2 × β_Factor2 _+ Factor3 × β_Factor3 _+ Factor4 × β_Factor4 _+ Factor5 × β_Factor5 _+ constant (-.761)

If p results in a value smaller than .5, it is assumed that the patient will not use counselling. If p is higher than .5 it is assumed that counselling will be used.

The values for the different factors can be computed by using Table 2. The individual value for example in the burden scale for family carers has to be multiplied with .838 for Factor 1. But this is only part of Factor 1. In order to compute Factor 1 completely all individual values for the variables named in Table 2 have to be multiplied with the coefficient corresponding to the respective factor and than subsumed. In this way the individual Factors 2, 3, 4 and 5 can also be computed.

## Discussion

The special feature of the Counsellor Contact Caregiver (CCC) concept is that the family physician recommends early family counselling to family members caring for patients with dementia, and the counsellors actively contact these family caregivers by telephone. This form of "approaching" family counselling in dementia has not yet been empirically investigated. The usage of more extensive counselling with more than one personal contact is tailored to the individual needs of the family members and corresponds to a realistic care situation so that the study results can be transferred to existing care structures. This is especially important, as there are no widespread specialised dementia care services in Germany,

Without mediation of family counselling, as made here by the GPs, 6% of family caregivers in Germany contact a family counselling service at some point while caring for a patient at home. A usage rate of less than 10% was found even for family members of patients with dementia in a study with 151 patients, of whom 78% suffered from mild to moderate dementia [10]. Comparison of several European countries shows a usage of counselling services of 4 to 5% [7]. In the present study, usage could be increased several times over by arrangement through the family physician and by establishment of initial telephone contact by the counsellor. More extensive family counselling with more than one personal contact within a 12-month period was used by 36% of the family members. This must be taken in the context that the family members involved are family members of patients with mild to moderate dementia, so the aim of **early **counselling could be successfully implemented.

As predictors for usage, it was found that caregiving spouses and family members for whom the care led to greater subjective and objective burden used the offer of personal counselling contacts more frequently. Knowledge of the predictors for using a counselling service makes it possible for the counsellors to adapt specifically to the needs of this target group. Since there is an increased general morbidity risk with increasing age and the spouse generally has a greater burden of care and poorer quality of life than other family members, it is particularly important to reach this "risk group" with a counselling service [24]. Moreover, the extent of emotional stress for the caregiving family member must be taken into consideration especially in the care of patients with dementia, since there is evidence of an elevated risk that the family member will become ill (e.g. depression) and that the risk of inappropriate care behaviour increases with increasing subjective stress [25,26].

How many and especially which family members in fact make use of counselling intervention is seldom addressed in studies. For the REACH program study centre, Miami, with a structural family therapy intervention with 75 participants, a mean contact frequency is cited (13 times within one year). However, there is no mention made of how many and which family members really "go along" with the intervention, that is the difference between "users" and "non-users". Since about 36 sessions per caregiver were planned, it can be concluded that some of the family members did not participate in the intervention [27]. In another large US-American study with 203 caregivers in the intervention group recruited in a memory clinic, there was first a fixed "counselling scheme" of 2 individual and 4 family counselling sessions, in which all caregivers had to participate, followed by an open service in which the counsellor was available on request. No presentation was made of which family members actually made use of the open counselling service [28].

In the present study, the contents discussed in the personal counselling contacts were "physical situation of the patient" and "general framework". Thus it was found that especially the patient's physical situation and the changes resulting in the need for care with respect to provision of medical and supportive aids, suitable adaptation of housing and financial situation were important in the counselling sessions. The needs of the family members, namely their own emotional situation, were less often in the middle point of the sessions. If the counselling wishes of family members are taken into account, favourable effects can be expected. Newcomer and colleagues were able to demonstrate that specific inclusion of the topic "clinical nursing and caregiver support activity" was even positively associated with a lower rate of hospital admission of the patient [29].

The results of the present study are based on a sample of 97 patients with dementia and their family members, who were treated by 46 general practitioners. The usage of family counselling could be clearly increased by CCC. However, 30% of the family members also had no personal contact and 34% only one personal contact with the counsellors. It must be taken into account that these are family members of patients with mild to moderate dementia, among whom it can be assumed that some of the family members really do not yet have a need for counselling. Nevertheless, also for these caregivers it might helpful to know where to seek help in case it is needed in future.

As a limiting factor no details of the contents of telephone contacts are available, therefore the question of the extent to which qualitatively meaningful counselling contact was made here must remain open. Certain counselling contents, such as information about the nearest nursing service, can be effectively presented by telephone. Nevertheless, sensitive and complex topics, such as dealing with a dementia patient's demanding behaviour, require personal contact if relief is to be achieved for the family member [13]. The significance and extent of resulting effects of CCC must be addressed in future studies. Furthermore it would be interesting to apply CCC to caregivers of patients with non-cognitive disorders.

## Conclusion

The investigation presented here shows that approaching family members actively is one way of establishing family member-counsellor contact early on. For practice, this means that in family medicine, attention should be paid to those family members, who are the main caregiver of a patient, especially when they perform a high number of hours of care and are subjectively burdened. In these cases, it would be desirable if the general practitioners - with the family member's permission - were allowed to provide family member's contact data to family counselling services on a regular basis. The counselling service could then offer an early support for these caregivers.

## Competing interests

The sponsors have commissioned two academic research institutes with the scientific evaluation of the IDA project by giving unconditional research funds. A contract between the sponsors and academic researchers ensures that the latter have full scientific responsibility and the right to publish the results. Members of the sponsoring organisations cooperate closely in the design and conduct of the project, but only the academic researchers have full access to all of the data in this study and take complete responsibility for the integrity of the data and the accuracy of the data analysis.

MGS, CD, RH and EG are independent scientists who have received funding for this study as described above. JL, SR, HM, PM and SW are or have been employees of the sponsoring institutions.

## Authors' contributions

MGS participated in the local coordination of the study and drafted the manuscript. CD participated in statistical analysis and interpretation of the data. RH participated in the design of the study and contributed in the statistical analysis. JL, SR, HM, PM, SW conceived of the study, participated in its design and coordination and helped to draft the manuscript. EG is principal clinical investigator, participated in the design of the study, and revised the manuscript substantially. All authors contributed to the manuscript and have read and approved the final version of the manuscript.

## Pre-publication history

The pre-publication history for this paper can be accessed here:

http://www.biomedcentral.com/1471-2318/10/24/prepub

## Supplementary Material

Additional file 1 **Table S1 - Guideline for CCC**. Word Document containing a table which describes the guideline for "Counsellors Contact Caregivers" (CCC) including risk factors for institutionalization and corresponding measures Click here for file
