# Supplementary material for: Counsellors contact dementia caregivers - predictors of utilisation in a longitudinal study
Source: BMC Geriatr. 2010 May 14;10:24. doi: 10.1186/1471-2318-10-24 (PMC2882905; doi:10.1186/1471-2318-10-24)
Supplement: Additional file 1 — Table S1 - Guideline for CCC. Word Document containing a table which describes the guideline for "Counsellors Contact Caregivers" (CCC) including risk factors for institutionalization and corresponding measures [file 1471-2318-10-24-S1.DOC]

| Risk factor: **Table S1: Guideline for “Counsellors Contact Caregivers”**  **Documented facts** | Evidence base of risk factors of nursing home placement | Prioritised measures taken by the counsellor |
| --- | --- | --- |
| **Primary caregiver alone** | Primary caregiver is sole support person (positive, if family helps) [1-3] | Involve social environment as much as possible, see "General measures" (at the end of the table), propose using whatever easily accessible services are available (e.g. help groups, family caregiver support groups, neighbourhood help) |
| **Primary caregiver's health impaired** | Primary caregiver's health, subjective health, changes in own IADLs, caregiver's age (> 65) [3-5] | Encourage seeking medical advice. Counselling to increase awareness of own physical and mental health problems, strengthening self perception and addressing preventive measures (e.g. physiotherapy, relaxation exercises, nutrition counselling).Help to get housing advice, if necessary |
| **Primary caregiver has little social support** | Dysfunctional family (other relatives have a higher risk than married couples), little social support [2, 6-10] | Talks about reducing burden of care and resolving conflict. Multi-person counselling.Encourage and help to get cognitive social support (e.g. outside counselling) emotional social support (e.g. family caregiver support groups) and practical social support (see "General measures"). Counselling on recognising and developing own interests. |
| **Primary caregiver requires more help with nursing** | Evidence of later entrance to nursing home when taking up the offer of: early use of „community-based services“, day care, home nursing services [3, 11, 12]  Evidence of earlier entrance to nursing home when these factors are present: extent of knowledge about dementia generally, short-term care, need of more specialist care, accommodation at home not suitable _1_, [4, 7, 13] | Point out what is available in the region, (see "General measures"), make suggestions how to use them as needed. Tailored counselling on demand (Aim: to improve competence in domiciliary nursing), counselling about adapting the living environment, address availing of complementary out-patient care (e.g. day care, neighbourhood help). |
| **Primary caregiver overtaxed** | Subjective burden of caregiver (depression, quality of life, distress) [2, 3, 5, 6, 8, 13-16] | Raising subjective well-being by having psycho-social talks to relieve the burden of care, offering grief counselling (step by step loss of close relative's personality through dementia). Addressing the changing role within the relationship. Help to get outside counselling or other treatment as necessary, (e.g. medical care, special self-help groups). Addressing preventive measures (e.g. strategies for dealing with stress, dealing with conflict), help in developing individual strategies for coping. Help to get direct relief (see "General measures"). Offer a closing counselling session in the case of death |
| **Planned or unplanned absence of primary caregiver** | Absence of primary caregiver (planned/unplanned) | Discussing outside care facilities in advance (e.g. relations, professional services). Advice about financial support. Organising a surrogate caregiver (e.g. important telephone numbers, daily routine, medication regime, patient's likes and dislikes). |
| **Patient with markedly disturbed behaviour** | Abnormal features, (in particular physical aggression, hallucinations, depression) [1, 2, 4, 5, 10, 13, 17] | Specific knowledge counselling: counselling and information about various abnormal behavioural features. Suggest a specialist examination. |
| **Patient: Dementia getting worse** | Severity of dementia [1, 5, 14, 17, 18] | Mild and moderate stage: Motivate relatives to activate patients to preserve existing faculties and promote patient's interests; Create feeling of success for patients (e.g. by doing housework, going for a walk together). Suggest a specialist examination with therapy trial (neurological consultation through the GP). Suggest a non-medication-based therapy (e.g. physiotherapy). Knowledge counselling (e.g. how to deal with dementia patients). Procedure counselling (help to self-help). |
| **Patient: needs more help with ADLs, physical degeneration** | Help required with one or more ADL or functional status (Barthel), deterioration of ADL, incontinence,  General physical degeneration (fall, other medical risk factors such as weight loss, dehydration, deterioration of sight and hearing, side effects of medication, "hidden" pain where agitation is present) [3, 5, 7, 10, 14, 17] | Knowledge counselling (e.g. information about level of care diary) Domiciliary nursing support, housing advice if necessary(see "General measures"). Providing medical appliances / apply for more domiciliary nursing services. Recommend specific information (e.g. brochures: „Incontinence in dementia“, "Food and drink for dementia sufferers"). Addressing preventive measures (e.g. prevent falls, hip protectors, pressure mattress, correct nutrition and hydration, get hearing tested, get eyes tested, if necessary encourage geriatric consultation (accident prevention) |
| **Patient: Planned or unplanned stay in hospital** | Patient in hospital | Inform in advance that increased counselling is available during a stay in hospital. Try to maintain intensive contact with the family at this time. Offer to make contact with the ward or hospital social services. Help to organise domiciliary care (e.g. preventative care, domiciliary nursing service) |
| **"General Measures" consist of:** Informing about domiciliary available nursing services in the region and helping to get them, nursing training, care counselling, out-patient ancillary services, 24-hour care at home, day care, family caregiver support groups, home help, care groups, neighbourhood help, voluntary helpers, self-help groups, sheltered communal living with warden, community service workers, supervision by the hour, support from religious or Church congregations, housing advice. | | |

**References**

1. Gilley D, Bienias J, Wilson R, Bennett D, Beck T, Evans D: **Influence of behavioral symptoms on rates of institutionalization for persons with Alzheimer's disease**. *Psychological Medicine* 2004:1129-1135.

2. Banerjee S, Murray J, Foley B, Atkins L, Schneider J, Mann A: **Predictors of institutionalization in people with dementia**. *Journal of Neurology, Neurosurgery and Psychiatry* 2003, **74**(9):1315-1316.

3. Gaugler J, Kane RL, Kane RA, Clay T, Newcomer R: **Caregiving and institutionalization of cognitively impaired older people: utilizing dynamic predictors of change**. *Gerontologist* 2003, **43**(2):219-229.

4. Buhr G, Kuchibhatla M, Clipp E: **Caregivers' reasons for nursing home placement: clues for improving discussions with families prior to the transition**. *Gerontologist* 2006, **46**(1):52-61.

5. Yaffe C, Fox P, Newcomer R, Sands L, Lindquist K, Dane K, Covinsky K: **Patient and Caregiver Characteristics and Nursing Home Placement in Patients With Dementia**. *JAMA: the journal of the American Medical Association* 2002, **287**(16):2090-2097.

6. Spitznagel M, Tremont G, Davis J, Foster S: **Psychosocial predictors of dementia caregiver desire to institutionalize: caregiver, care recipient, and family relationship factors**. *Journal of geriatric psychiatry and neurology* 2006, **19**(1):16-20.

7. Spruytte N, Van Audenhove C, Lammertyn F: **Predictors of institutionalization of cognitively-impaired elderly cared for by heir relatives**. *Int J Geriatr Psychiatry* 2001, **16**(12):1119-1128.

8. De Vugt M, Stevens F, Aalten P, Lousberg R, Jaspers N, Verhey F: **A prospective study of the effects of behavioral symptoms on the institutionalization of patients with dementia**. *Int Psychogeriatr* 2005, **17**(4):577-589.

9. Pot A, Deeg D, Knipscheer C: **Institutionalization of demented elderly: the role of caregiver characteristics**. *Int J Geriatr Psychiatry* 2001, **16**(3):273-280.

10. Thomas P, Ingrand P, Lalloue F, Hazif-Thomas C, Billon R, Vieban F, Clement J: **Reasons of informal caregivers for institutionalizing dementia patients previously living at home: the Pixel study**. *Int J Geriatr Psychiatry* 2004, **19**(2):127-135.

11. Gaugler J, Kane RL, Kane RA, Newcomer R: **Early community-based service utilization and its effects on institutionalization in dementia caregiving**. *Gerontologist* 2005, **45**(2):177-185.

12. Hirono N, Tsukamoto N, Inoue M, Moriwaki Y, Mori E: **Predictors of long-term institutionalization in patients with Alzheimer's disease: role of caregiver burden**. *No to Shinkei Brain and Nerve* 2002, **54**(9):812-818.

13. Whitlatch C, Feinberg L, Stevens E: **Predictors of institutionalization for persons with alzheimer's disease and the impact on family caregivers**. *Journal of Mental Health and Aging* 1999, **5**(3):275-288.

14. Rozzini L, Cornali C, Chilovi B, Ghianda D, Padovani A, Trabucchi M: **Predictors of institutionalization in demented patients discharged from a rehabilitation unit**. *Journal of the American Medical Directors Association* 2006, **7**(6):345-349.

15. Gilley D, McCann J, Bienias J, Evans D: **Caregiver psychological adjustment and institutionalization of persons with Alzheimer's disease**. *Journal of aging and health* 2005, **17**(2):172-189.

16. Nobili A, Riva E, Tettamanti M, Lucca U, Liscio M, Petrucci B, Porro G: **The effect of a structured intervention on Caregivers of patients with dementia and problem behaviors - A randomized controlled pilot study**. *Alzheimer Disease & Associated Disorders* 2004, **18**(2):75-82.

17. Strain l, Blandford A, Mitchell L, Hawranik P: **Cognitively impaired older adults: risk profiles for institutionalization**. *Int Psychogeriatr* 2003, **15**(4):351-366.

18. Hébert R, Dubois M, Wolfson C, Chambers L, Cohen C: **Factors associated with long-term institutionalization of older people with dementia: Data from the Canadian Study of Health and Aging**. *Journals of Gerontology Series A, Biological Sciences and Medical Sciences* 2001, **56A**(11):M693-M699.
